# Identity challenges and ‘burden of normality’ after DBS for severe OCD: a narrative case study

**DOI:** 10.1186/s12888-018-1771-2

**Published:** 2018-06-13

**Authors:** Peter Bosanac, Bridget Elizabeth Hamilton, James Lucak, David Castle

**Affiliations:** 1 St. Vincent’s Hospital, Melbourne and Department of Psychiatry, University of Melbourne, Melbourne, Australia; 2 School of Health Sciences, University of Melbourne, Melbourne, Australia; 3 St Vincent’s Hospital, Melbourne, Australia

**Keywords:** Anxiety, Obsessive-compulsive disorder, Deep brain stimulation, Adjustment, Burden of normality

## Abstract

**Background:**

Deep Brain Stimulation (DBS) is an emerging and potentially powerful biological treatment for severe Obsessive-Compulsive Disorder (OCD), but the wider impact of the intervention and the sometimes dramatic reduction in symptoms need greater attention in research and practice. The aim of this case study is to explore the subjective experience of preparing for and undergoing DBS as a treatment for severe and treatment-refractory OCD and the experience of the impact of the treatment.

**Methods:**

This study of subjective experience before and after DBS is based on narrative analysis of two in-depth interviews conducted in November 2014 (1 year after DBS surgery) with a 30-year-old man and his father, utilizing Consolidated Criteria for Reporting Qualitative Studies (COREQ) criteria.

**Results:**

The parallel stories show how OCD posed severe challenges to identity and social milestones, with profound positive and negative impact on the person and family. Yet symptom remission was accompanied by expanded horizons, but also by uncertainty and intense distress associated with the changed identity.

**Discussion:**

The concept of ‘burden of normality’ is discussed, in light of a treatment experience with DBS for OCD that gives rise to a new array of life challenges and opportunities, with implications for clinical care.

**Conclusions:**

The concept of burden of normality has, thus far, not extended to evaluations of people who have had DBS for severe OCD and that of their lived experience and recovery trajectory thereafter. This concept highlights that there is work to be done on expectations of normal living and on the transitioning self-concept, in the post-surgical period.

## Background

Obsessive-compulsive disorder (OCD) is a chronic psychiatric disorder that affects 2% of the general population [1, 2]. Despite therapeutic trials of evidence-based treatments for OCD, such as cognitive-behavioural therapy with exposure and response-prevention, serotonin re-uptake inhibiting antidepressants and augmentation with second-generation antipsychotic medications or newer glutamatergic agents [3], approximately 30–40% of people with OCD have a poor treatment response and 10% remain severely affected (treatment-refractory) [1, 2]. Deep Brain Stimulation (DBS) for people with severe and treatment-refractory OCD, for which there is a humanitarian exemption in the USA, has largely been contextualized within the clinical research domain [4], albeit with greater, emergent meta-analytic and systematic evidence for its consideration and use in this clinical population [5–7].

While remission of symptoms following DBS may be welcomed, changes to perceived identity and relationships can also be profound. Recovery is well recognised as a non-linear process that people are engaged in, as they form a life and an identity beyond psychiatric illness [8]. Since Bury’s [9] sociological work with arthritis patients, the experience of biographical disruption is widely reported, in regard to the life impacts following emergence of and adjustment to a range of chronic illnesses. In regard to psychiatric illness, researchers have identified how illness, associated treatments and social context disrupt life and shape identity [10]. Consequently, the concept is not solely tied to curative experiences, after a long illness experience. The burden of normality (defined as difficulty in adjusting to being free of significant symptomatology) has been evaluated in neurological conditions such as epilepsy. The burden of normality following DBS for neurological disorders [11, 12] highlights the importance of taking into account personality and illness duration to address the period post DBS. Literature amplifying consumer voices in regard to health, illness and treatment has grown steadily in recent decades, with recognition of the centrality of consumer views to development of evidence-based practice. But, the implications of DBS for the lived experience of the individual, including sometimes dramatic reduction in symptoms, have only recently been appreciated [13, 14]. A study of 18 participants aged 26–65 years following DBS for severe and treatment-refractory OCD explored their broader post-operative experience via semi-structured interviews, beyond that of changes in obsessive-compulsive symptomatology alone [14]. The study found that overall, participants reported experiencing increased trust, self-reliance and confidence, as well as being more carefree and impulsive, less preoccupied about their circumstances, and improvement in mood and the extent of anxiety. However, the interviews were conducted 6 to 91 months following DBS, with an increased possibility of recall bias, including that of some nuances of subjective experience. Also, the study did not include participants with extremes of therapeutic response, with none of those being interviewed having almost complete response in their obsessive-compulsive symptoms; the latter reportedly declined participation in further research, opting to just get on in regard to their lives.

Whilst the collaborative and coproduction involvement of mental health consumers in their care planning endeavours to, and may improve their esteem and optimize the development of services and the attitudes of clinicians providing these [15], there are significant barriers such as the divergence of the consumer’s frame of reference with that of the service provider [16]. Moreover, consumers and carers attribute the greatest value to relational aspects of care planning, albeit with inconsistent consideration by service providers [16]. Unfortunately, the richness of mental health carers’ perspectives are suboptimally utilized [17].

This paper presents a case study of the subjective experience of a young man with severe and treatment-refractory OCD, preparing for and undergoing DBS, in which the profound psychological and social impact on his life and that of his family are explored following marked remission of his illness symptoms.

## Methods

A case study was proposed by the participant with DC and PB. This arose from DC’s and PB’s experience in the clinical setting, in context of the participant being the first to have DBS implantation for OCD at the health centre, and with the view of exploring the participant’s lived experience and that of those closest to him.

The case study was produced through a narrative analysis of two, 120 min, in-depth interviews with a patient Mr. A and his father in November 2014. The methods are described with reference to elements of the Consolidated Criteria for Reporting Qualitative Studies (COREQ) [18] criteria and checklist that apply to qualitative case study. The case study was designed around the experience of DBS from the points of view of two people who were known (by DC) to be key to the decision to undergo DBS and who could speak powerfully about the impact of symptoms and treatments across the consumer’s life course. Interviewing both the consumer and the primary carer/father was designed to gather a rich narrative account through several key times in life-course: early life and development of severe OCD, the decision making for DBS and the recovery in the aftermath of the intervention. Member-checking of the narrative analysis with the participants enabled the interweaving of these subjective experiences into one coherent narrative.

### Research team and reflexivity

Mr. A was the first participant in the DBS for severe and treatment-refractory OCD study conducted at the centre (Australian and New Zealand Clinical Trial Registry identification: ACTRN12612001142820). Mr. A was known clinically to DC, and to PB via assessment for, and participation in the study. The Human Research Ethics Committee reference for the DBS study was HREC/12/SVHM/64.

DBS uses a surgically implanted, battery-operated medical device called a neurostimulator (implanted subcutaneously under the collar bone, or elsewhere) to deliver electrical stimulation to targeted areas in the brain (guided by 3 dimensional stereotactical magnetic resonance imaging) and micro-electrical recordings to help with differentiation of the targeted area, namely the nuclei accumbens in the deep basal part of the forebrain, from other areas of brain) via implanted electrodes inserted through small apertures in the skull. Participants are awake during most of the procedure (with local anaesthesia used to create the apertures in the skull) up to several hours, but requiring a general anaesthetic for the implantation of the neursostimulator and the extensions under the skin in the neck connecting this to the where the electrodes enter the skull. The electrode is inserted through a small opening in the skull and implanted in the brain. The study in which Mr. A participated, consisted of 3 phases (‘acute’ phases 1 and 2- fortnightly reviews for 9 months with adjustment of neurostimulion parameters via a programming device applied directly over the site of the neurostimulator, and baseline and 9 month neuropsychological tests and positron emission scanning of the brain, as well as cognitive-behavioural therapy depending on the extent of symptomatic response, and occupational therapy support; and a ‘maintenance’ phase 3, with 3 monthly follow-up, including review and adjustment of neurostimulation parameters).

Mr. A had reported his wish to share his experience of DBS and their “burden of normality” with those considering and being considered for DBS for OCD, so that they could learn from his experience and better able to provide informed consent. Mr. A had also explored literature available at the time on the “burden of normality” and first approached DC, who in turn approached PB. BH made contact initially via telephone. Interviews for the case study were conducted by BH, a female academic nurse (no other reported interviewer characteristics) with mental health expertise, an extensive background in qualitative research and a PhD. The interviewer had no prior clinical relationship to or contact with the participant or family member.

### Study design

The narrative interviews were conducted in November 2014, 1 year after DBS implantation, and were designed to elicit rich, chronological accounts of the lived experience of OCD and DBS, from the interwoven perspectives of son and father. This was in context of the participant being the first to have the DBS procedure at the health service, as well as gaining a broader and more holistic perspective of the impact of the procedure on the participant and their main carer. The interview questions included: *In order to understand your experience of OCD and DBS, where do we need to start? What happened next? When did things change for you?* The interviews were conducted and audio recorded at university premises, and transcribed by BH. Conduct of this study was supported by the Research Governance Office of St. Vincent’s Health, with advice that no formal ethics approval was required by the Human Research Ethics Committee at St Vincent’s Hospital, Melbourne, in this particular case, but that the case study be in in line with ethical standards (applicable privacy guidelines and legislation).

### Analysis

The chronological accounts were analysed by BH. Narrative analytic method was informed by the narrative schema of Labov and Waletzky [19]. The interview of Mr. A was analysed first for narrative form, highlighting setting, characters and their motivations, plot, dramatic moments, and resolution points. Then the story arc was further enriched by analysis of the story elements and points of view elaborated by his father. There was no other frame or software used for data coding. Data saturation was not relevant to the study design.

A process of engaging Mr. A and his father in review of the data and analysis was undertaken, to ensure the validity of the research outcome. After a full draft was constructed, meaning and detail in the analysis was refined and confirmed, through careful review with both participants. Mr. A and his father’s quotations were utilized to demonstrate narrative themes.

## Results

### Case study

Mr. A is the younger of two children born in Australia to parents with European migrant heritage. His life took a significant turn for the worse during later childhood, when his OCD first manifested. Mr. A recalls himself as an energetic and outgoing child, but his experience shifted from unselfconsciously living, to feeling increasingly uneasy in his body and social world. The following narrative explains key decision points and psychosocial impacts of DBS for severe OCD in this case, from the subjective perspectives of Mr. A and his father.

### ‘The trouble’

From his late teens, ritualized checking behaviors increasingly dominated Mr. A’s family- and home- life, eroded his school participation and intimate relationships and then cost him his potential opportunities for work, noting that after initially and briefly commencing tertiary studies, he had not been previously employed. After Mr. A received an OCD diagnosis at 16 years, the family determinedly pursued recommended treatments. Despite following the advice of experts in the field, taking prescribed medications and engaging with many cycles of intensive inpatient and community care, a series of new and overwhelming obsessive thoughts emerged, intermingled with new and extensive rituals of checking and washing. His social world narrowed to the family home, where he interacted only with family members and online in highly competitive video gaming.

Mr. A’s father reflected on his sense of helplessness through Mr. A’s adolescent period: *“I would have to sit quietly, sometimes an hour, until he did it [adjusted objects in the house] and he would get himself completely worked up. There was sweat coming off of him and, you know, he’d change his clothes. … I think the really sad part was that I was watching my son become an ‘invalid’ in his own – he was a prisoner of his own mind, in his own room and there was nothing – no medication or anything that I could, you know, do to help him.”*

### ‘The search’

In the mid-2000s, after a hospital admission and continuous months of confinement at home, Mr. A and his father found information online about DBS, including reports of its use for OCD. His father says: *“That then sort of set us on a course of, ‘Let’s see if we can get this done’, because he’s at the point where he couldn’t function.”* They were both attracted to this possible solution, but initially found no support for the idea among local mental health professionals, as Mr. A describes: “*I asked them, I said, ‘Look, is there any chance I can get deep brain stimulation?’ and they laughed at me and they said, you know, ‘There’s no way they’ll operate on your brain.’”*

Mr. A and his father wrote emails to several psychiatrists, saying: “*We want somebody thinking out of the box to look at DBS and other drug therapies*”, and found a psychiatric team who would consider DBS. There were protocols and ethical reviews to follow, before such surgery was possible. A year later, Mr. A worked with exposure therapy to overcome a final hurdle that they both recognized as significant: his fear of being touched on his head. Mr. A’s determination to pursue DBS was evident, as he confronted excruciating anxiety, in order that he could tolerate being touched, in preparation for surgery.

### ‘The turning point’

Mr. A’s father recounted a precise moment in the consulting rooms, 3 days post DBS surgery: *“When he [the doctor] turned ithe DBS] on …, [Mr A] felt something different in his mind at that particular time and when the doctor went to switch it off, [Mr A] said, ‘Please don’t. It’s the best I’ve ever felt,’.*” Likewise, Mr. A himself described an immediate change in his OCD feelings and thinking. He recalls saying to the supervising psychiatrist: “*Look, I really want this on constantly”.*

Over the following days, weeks and months, Mr. A enthusiastically challenged a great list of behaviors and rituals that had filled his life for more than a decade. His father recalled: “*In this period of time he became very – like, ‘I want to do things, very hyper.*” With coaching from an Exposure and Response Prevention outreach worker, Mr. A made great strides in eradicating OCD features from his life. He also broadened his life, beginning volunteer work in a school and then an aged care centre. After 9 months, he could say: *“I don’t have any rituals left. So I consider that as a full recovery. And my symptom score would probably be a zero, you know.*” Through this time, his father also experienced a sense of freedom, without the daily constraints of Mr. A’s rituals: “*I felt freer, you know, and things started to move ahead*”. As he made progress, he pursued three goals: “*One was to, you know, volunteer. One was to get into uni and one was to find a girlfriend*.”

### ‘New troubles’

A crisis of a different sort struck 9 months after DBS surgery, when Mr. A was finally able to take a shower, an activity that had been fraught for many years. This signified a final OCD obstacle for Mr. A. He experienced unexpected turmoil of emotions: *“But once I did that last thing, I was then without OCD. So I was a new person…So my whole life I was ‘sick’, you know, and then I became ‘What the hell am I now?’ And it was very upsetting. I would often cry in front of dad and say, ‘I don’t know what to do.’*” Such concerns had not surfaced post-DBS and while he was focused on defeating OCD. But with wellness came existential questions about purpose. *“Once I became, you know, 100 per cent better I went through all of this turmoil of what to do, you know. Do I volunteer anymore? Do I go to uni anymore? What the hell do I do?”*

The way his father explained this turmoil was to see Mr. A’s development as suspended through OCD and now as re-starting: “*I’d lost my son in adolescence. This is 15 [gesturing at one point on the table edge]. … he’s 30 now [pointing further along the table edge]. To me, he’s just starting here [pointing back at the 15 year mark]”.*

Mr. A also perceived himself as lacking skills for living well: “*I was confronted with all these new feelings and not knowing how to react to things, being well, like – there was a set way that I’d react to situations when I had OCD. I knew what I would do. But now that I’m well, when a situation comes up, I don’t know how to react*”. He reflected that OCD had filled his daily life and thoughts for so long, crowding out other aspects of living.

Mr. A felt unprepared for this mixed experience of uncertainty and exhilaration at being OCD free: *“Well, where was my two years of, you know, preparing me for a new identity? There was no - you know, it was bang, ‘Here’s your new identity, just deal with it yourself.’ I mean, I had no preparation. I had no pre-warning that it might happen.”* Mr. A’s father affirms the depth and breadth of change for Mr. A: “*from being not able to do anything – even his appearance, the way he talked, everything changed*.”

### ‘The learning’

Mr. A was both proud of his recovery and also dismayed, at having to cope with change that was so drastic, giving rise to a new set of problems. This sense persisted to the time of interviews, a year after the DBS surgery, though it was less potent. He likened the identity challenges he experienced to rebirth: “*It’s like, you know, you’re born again, like – it’s like being born for the first time.*” He wondered if the treating team could have prepared him better for this phase of life. His father agreed that there could be more support for a person experiencing such major adjustment: “*I think it’s a loss of one particular part of your life that you’ve had for a long period of time and then the not knowing, what do I do, to make it work now...*”

Mr. A also reflected: *“There’s that line of recovery and …you need to make a pretty big recovery to actually get to this point where you’re a new person and you have a new identity. You need to shed your whole illness. You need to get rid of it completely, which is what I did, which was phenomenal like, you know – the guy running the trial was like – was really impressed, you know, every time I came, you know, I was like, “Okay. It was fine, fine, fine,” but that ended – when all the testing [was fine] and that’s when I got these problems.”*

Mr. A was aware that his baseline Yale-Brown Obsessive-Compulsive Scale (YBOCS) [20] score, a quantitative measure of the severity of obsessions and compulsions, was 30 prior to DBS, which is in the severe range. Eight months after the DBS procedure, his score was 6, which is in the sub-clinical range, where it had approximately remained for over 2 years.

## Discussion

This case study highlights the biographical disruptions that can be associated with development of chronic illness, symptom experiences over time and also with changes associated with treatment. The first-person details here convey the grinding imposition of OCD, endured by Mr. A and his father. This experience explains the decision to pursue a neurosurgical solution such as DBS. For the treating team and for the patient, the almost complete resolution of symptoms post DBS was very striking. But Mr. A and his father also grappled with the memory of wasted years and the ‘burden of normality, as not being directly familiar of life as a reportedly ‘normal’ person.

As we see in this case, the ‘burden of normality’ can arise when a transition in self-concept occurs, associated with the shift from chronic disability to sudden wellness [21]. Where this transition is accompanied by distress, this is in terms of the individual and family expectations of normality that will span a range of complex psychological and social issues, depending also on the perceived effects of a chronic disorder. There may be a mismatch between the longed for expectations and actual experience of normal life, as is described by epilepsy researchers [11]. The shortfall in skills in understanding oneself and relating to the others and world in general may be directly due to the disability or may reflect some other lack, including limited developmental opportunity, as in the case of Mr. A.

It is noteworthy that in this case Mr. A’s reported distress arose only once a final OCD symptom was resolved. Resolution abruptly generated a large and unexpected void in the life of Mr. A, as a young man. This observation accords with the theoretical explanation burden of normality in terms he resolution of symptoms and affected functioning attributed to OCD that it is driven by the expectations held by individuals about a disabled self and a normal self [11, 22]. Distress was not experienced while Mr. A still had recovery work to do; a crisis of identity surfaced only when ‘normality’ was reached.

Mr. A’s father explained the turmoil in terms of Mr. A suddenly confronting a backlog of developmental tasks, independent of the issue of disability. This observation directs our attention to the possibility that the burden of normality concept may have particular application to adolescent and young adult populations experiencing abrupt relief from a debilitating illness [11].

A final point is well-made by A and his father - that DBS recipients and their families may benefit post-surgery from peer support (lived experience of recovery that comes from firsthand experience and may not be understood in such a nuanced or holistic way by health professionals without this firsthand experience) [22], even to adjust to wellness. For both these people, the focus now is on the future and helping Mr. A regain his life, in this important phase of Mr. A’s journey. Clinicians need to be aware of the potentially distressing impact of abrupt ‘liberation’ from what has been referred to as ‘the tyranny’ of OCD [23] so they can mitigate distress by assisting with the transition.

The strengths of this narrative include the extent of information sought from Mr. A about the significance and context of DBS surgery and follow-up as a part of a clinical research study in terms of their lived experience of OCD, their individual recover journey and the impact of the ‘ burden of normality’ for them. Moreover, the exploration of the ‘burden of normality’ is novel ground in this clinical population. However, limitations of this study include the potential generalization to other consumers having DBS for severe treatment refractory OCD may not be generalizable, as recovery pertains to an individual’s trajectory, Mr. A was highly motivated to share his experience of DBS and subsequent challenges with those considering DBS for OCD, and individuals electing to undergo DBS in this clinical population will experience differing degrees of symptomatic response. Moreover, interviewing both participant and carer may have impacted on the process and content of the narrative study in terms of their father-son relationship and what they may have been able to describe narratively whilst being interviewed together. Hence, the narrative study setting could have been enhanced by interviewing them individually, as well as together. Also, the current study (or future studies) would be enhanced by researchers reflecting on how their own backgrounds affect the findings and their analysis, although the data within the narrative was reviewed by the participant and their carer.

## Conclusions

Whilst the concept of burden of normality has been discussed to date in regard to experiences after treatment for neurological disorders, it has, thus far, not extended to evaluations of people who have had DBS for severe OCD and that of their lived experience and recovery trajectory thereafter. The burden of normality concept is helpful for highlighting that there is work to be done on expectations of normal living and on the transitioning self-concept, in the post-surgical period. 

## Appendix

### Narrative Interview Schedule re Case Study of DBS experience

This set of question provides a loose structure to facilitate telling of the experience before, during and after DBS, from each of the participants’ points of view.

*The interviewer is to use a series of narrative prompts as fitting with the flow of narrator’s speech:*

***Context for DBS***

When did the problems for (XXX participant) first emerge?

Then what happened? Then what? What was that like for you, for him, for the family?

What did you do? What did he do? How did you feel? What did you hope for? Who else was important in the experience?

***Experience of DBS***

*Narrative prompts if the interviewee’s account does not include events and thinking related to choosing DBS:*

How did DBS first come up for you (both)?

What did you hope for, expect?

What did you do? Then what happened? How was that for you?

***Life after DBS***

*Narrative prompts if the interviewee’s account does not include events and experience post-DBS:*

What happened for you (both) after DBS? What did you do?

What were your thoughts/feelings about it?

What else is important for me to understand about the whole experience, from you point of view?

## Abbreviations

COREQ: Consolidated Criteria for Reporting Qualitative Studies
DBS: Deep brain stimulation
OCD: Obsessive-compulsive disorder
YBOCS: Yale-Brown Obsessive-Compulsive Scale
